# Determinants of Preterm Birth among Women Who Gave Birth in Amhara Region Referral Hospitals, Northern Ethiopia, 2018: Institutional Based Case Control Study

**DOI:** 10.1155/2020/1854073

**Published:** 2020-01-08

**Authors:** Abebayehu Melesew Mekuriyaw, Muhabaw Shumye Mihret, Ayenew Engida Yismaw

**Affiliations:** ^1^Deneba Primary Hospital, North Shoa, Amhara Regional Health Bureau, Department of Midwifery, Ethiopia; ^2^Department of Clinical Midwifery, School of Midwifery, College of Medicine and Health Sciences, University of Gondar, Ethiopia

## Abstract

**Background:**

Preterm birth refers to a birth of a baby before 37 completed weeks of gestation and after fetal viability. It is now the leading cause of new born deaths. Although identifying its common risk factors is mandatory to decrease preterm birth and thereby neonatal deaths, there was a dearth of studies in the study area.

**Objective:**

The aim of this study was to identify determinants of preterm birth among women who gave birth in Amhara region referral hospitals, Northwest Ethiopia, 2018.

**Method:**

An institutional based case-control study was conducted from September 01 to December 01/2018. A total of 405 mothers (135 cases and 270 controls) were included in the study. Multistage sampling technique was employed. Data were collected using structured questionnaire through face to face interview and checklist via Chart review. Data were entered into Epi Info version 7 and export to Statistical Package for Social Sciences (SPSS) version 20 for analysis. Descriptive statics like mean, frequency and percentage was used to describe the characteristics of participants. Both bivariable and multivariable analyses were carried out. Variable having  *p*-value <0.05 in binary logistic regression were the candidate for multivariable analyses. Finally, the statistical significance of the study was claimed based on the Adjusted Odds Ratio (AOR) with 95% Confidence Interval (CI) and its *p*-value <0.05.

**Result:**

The result of multivariable analysis show that mothers with no formal education (AOR = 2.24; 95% CI: 1.28, 3.91), history of abortion (AOR = 2.92; 95% CI: 1.3, 6.4), multiple gestation (AOR = 4.1; 95% CI: 1.7, 9.8), hemoglobin level <11 gm/dl (AOR = 2.75; 95% CI: 1.11, 7.31), premature rupture of membrane (AOR = 6.4; 95% CI: 3.23, 12.7) and pregnancy induced hypertension (AOR = 4.74; 95% CI: 2.49, 9.0) had statistically significant association with experiencing preterm birth.

**Conclusion and Recommendation:**

Most of the determinants of preterm birth found to be modifiable. Thus, putting emphasis for prevention of obstetric and gynecologic complications such as anemia, premature rupture of membrane and abortion would decrease the incidence of preterm birth. Moreover, strengthening Information Communication Education about prevention of preterm birth was recommended.

## 1. Introduction

Preterm Birth (PTB), which refers to the birth of a baby before 37 complete weeks of gestation, poses multi-dimensional adverse consequences [1–9]. Globally, about 15 million (11%) of births are estimated to be PTB each year and about 12 million (over 81%) of these PTB occurs in Sub-Sahara Africa (SSA) and South Asia (SA) [10]. In Ethiopia, about 320,000 babies are born to soon and 24,000 Children Under Five Years (CUFYs) die of direct complication of PTB each year [11]. Likewise, the figure is high in Amhara National Regional State (ANRS) where 1 out of 8 (12.63%) of births reported to be PTB [12].

Prematurity causes enormous impacts [13]. It is now the leading causes of new Newborn Mortality (NM) and the second leading causes of Under-Five Mortality (UFM) preceded by pneumonia [14]. Moreover, it results in devastating complications which are responsible for 1 million children death each year. The implication of PTB is also extended beyond the neonatal period and throughout life. Babies who are born before maturity face the greatest risk of serious health problems including cerebral palsy, intellectual impairment, chronic lung disease, and vision and hearing loss. This adds dimension of lifelong disability. Most people will experience the challenges and possible tragedies of PTB at some point in their lives either directly in their families or indirectly through events for the countries [3, 8, 11, 15–18].

To alleviate this burden, efforts have been taken globally. For instance, Sustainable Development Goal (SDG) 3 target 2 is planned to end preventable death of new born and CUFYs [19]. In this regard, Ethiopia had made a remarkable achievement in infant mortality (IM) [20]. However, neonatal mortality (NM), which accounts 42% of UFM, has been declined slowly as compared to other child health indicators. To combat the setbacks, the national new born and child survival strategies has targeted to decreasing NM from 28% to 11% by 2020 [21]. Hence, to achieve these targets, better understanding of factors which predispose to NM, which is largely caused by PTB, is essential. To do so, conducting empirical investigations on major causes of NM including prematurity is crucial. Previous studies reveal that PTB has multiple factors such as previous premature birth, multiple pregnancies and short birth interval [22–24]. However, these factors may greatly vary across settings and time trends. In the study area, there are limited studies on predictors of PTB more particularly with strong study designs. Therefore, the current study is aimed at identifying determinants of PTB in the Amhara region referral hospitals.

## 2. Methods and Materials

An institutional based unmatched case-control study was conducted from September 1 to December 1/2018 in referral hospitals which is found in Amhara National Regional State (ANRS).

ANRS is one of the nine national states in Ethiopia which is found on the Northwestern part of Ethiopia. The region has 67 hospitals, 734 health centers, and 2941 health posts. There are 5 referral hospitals—namely Gondar University Comprehensive Specialized Hospital (GUCSH), Felegehiwot Comprehensive Specialized Hospital (FCSH), Dessie Referral Hospital (DRH), Debre-Markos Referral Hospital (DMRH) and Debre-Birhan Referral Hospital (DBRH). Each hospital serves for nearly 5 million people, poses 200–400 beds, and conducts 2000–4000 deliveries per year and 5–10 deliveries per day.

Three out of five of referral hospitals has been selected randomly using lottery method. These include; Felegehiwot Comprehensive Specialized Hospital (FCSH), Debre-Markos Referral Hospital (DMRH) and Debre-Birhan Referral Hospital (DBRH). All women who gave birth in the selected referral hospitals during the study period were taken as study population. Thus, all immediate postnatal women who gave live birth at the selected referral hospitals during the study period that had been included whereas those mothers who had neither certain Last Menstrual Period (LMP) nor early (i.e., at ≤20 completed weeks of gestation) ultrasound (U/S) evidences, had been excluded.

The sample size had been calculated through Epinfo version 7 by considering the following assumptions: ratio of control to case 2 : 1; power 80%; confidence level 95%, precision level 5%, Odds Ratio (OR) 3.81, design effect 1.5, nonresponse rate 10%, and percent of birth defect among controls (nonpreterm births) and cases (preterm births) 5.1 and 17 respectively from the study conducted in Tigray [25]. This provided a total sample size of 432 (which included 144 for cases and 288 for controls). Prior to the beginning of actual data collection, the sample size had been distributed to each selected hospital proportionally based on the previous deliveries' report. Thereafter, both cases and controls had been selected every three intervals by using systematic sampling techniques. The first participant was selected by lottery method among the first 3 cases and its corresponding controls were selected in a similar manner depending on the cases in such a way that the recruitment of controls basis the selection of their corresponding cases.

Socio-demographic, obstetric, medical, nutritional and fetal related variables had been incorporated. These include maternal age, residence, religion, maternal occupation, marital status, spouse occupation, maternal educational status, ethnicity, family size, spouse educational status, distances from home to nearest health facility, family monthly income, parity, inter-pregnancy interval, planned pregnancy, bad obstetrics history, Antenatal Care (ANC) visit, time of 1^st^ ANC visit, frequency of ANC, history of obstetric complications (Premature Rupture of Membrane (PROM), Antepartum Hemorrhage (APH), preeclampsia/eclampsia and poly/oligohydramnios) in the index pregnancy, history of preterm birth, multiple pregnancy, history of previous multiple pregnancy, history of abortion, iron provision, duration of iron provision, onset of labour, syphilis, Hemoglobin (HGB) level, history of chronic Hypertension (HTN), Urinary Tract Infection (UTI), Sexually Transmitted Diseases (STD) including HIV, Malaria, Mid Upper Arm Circumference (MUAC), maternal height, smoking, congenital anomalies, and birth weight. Moreover, cases (preterm births) and controls (nonpreterm births) had been selected based on the Gestational Age (GA) assessment.

The GA had been determined based on a certain Last Menstrual Period (LMP) date and/or early pregnancy ultrasound (U/S) established date (up to and including 20 completed weeks of gestation). When the LMP and U/S dates had not been correlated, defaulting to U/S for GA assessment was required in accordance with the American College of Obstetricians and Gynecologists (ACOG) recommendation [9, 26]. Those mothers with neither certain LMP nor early pregnancy U/S date for GA calculation had been excluded. Accordingly, births which had been conducted after 28 (fetal viability) and before 37 completed weeks of gestations were assigned to be cases (i.e. preterm births) whereas, those births which had been carried out after 37 completed weeks of gestation were allocated to be controls.

Data were collected using structured questioner and check list through face to face interview and chart review respectively. Questioner and checklists were developed after reviewing of various relevant literature [2, 27–30]. Interview and anthropometric measurements had been taken after delivery when the mothers become stable. In addition, client chart review had been undertaken by using check list to retrieve medical information and laboratory test which might not be captured by interview. A total of 11 health personnel had been involved in the data collection process. These included nine health personnel, who have diploma in Midwifery, as data collectors and three personnel, who own Bachelor of Science (BSc) Degree in Midwifery, as supervisors. Detail training on data collection process had been provided. Thereafter, pretest had been conducted on 5% of the sample sizes prior to the actual data collection commencement. During data collection period, necessary follow up and supervision had been undertaken, and the collected data had been checked for completeness, accuracy and clarity on daily bases.

Data were then entered in to Epi info statistical software version 7 and exported to SPSS version 20 for cleaning, recoding, categorizing and analyzing. Both descriptive and analytical statistical procedures had been utilized. Descriptive statistical like mean, frequency and percentage were used to describe the characteristics of participants and results presented using tables, graph and text. Binary logistic regression was used to identify factors associated with preterm birth. Finally, variables with *p*-value <0.05 were fitted to multivariable logistic regression model using Backward Stepwise method. Both Crude Odds Ratio (COR) and Adjusted Odds Ratio (AOR) with the corresponding 95% Confidence Interval (CI) were computed to show the strength of the association. Finally, statistically significant association of variables had been claimed based on the AOR with its 95% CI and *p*-value <0.05. In addition, model fitness had been checked using Hosmer and Lemeshow goodness of a fit test (*P* = 0.3).

## 3. Result

### 3.1. Sociodemographic Characteristics of Study Participants

In this study, about 405 mother-newborn pairs (135 cases and 270 controls) had been participated with the overall response rate of 94%. Majority of respondents (about 77.1% of cases and 84.1% of controls) were in the age group of 20–34 years with the mean (±Standard Deviation (SD)) of 28.6 (±5.4) for cases and 26.5 (±5.2) for controls. Most of the respondents (95.6% of cases and 91.1% of controls) were Orthodox Tewahido religion follower. Nearly all (97% of cases and 96.3% of controls) were belong to Amhara in Ethnicity. More than half (52.6%) of cases and one-third (35.2%) of controls were rural dwellers. In addition, about, 64 (47.4%) of cases and 70 (25.9) of controls had no formal education. Almost all respondents (99.3% of cases and 97% of controls) were in marriage relationship. More than four in five participants (84.4% of cases and 94.1% of controls) had total family size of ≤5. The time taken to the nearest health facility for most of the respondents (77.8% of cases and 141 89.3% of controls) observed to be ≤1 hour (Table 1).

### 3.2. Obstetric and Gynecologic Profile of Study Participants

**Figure 1:**
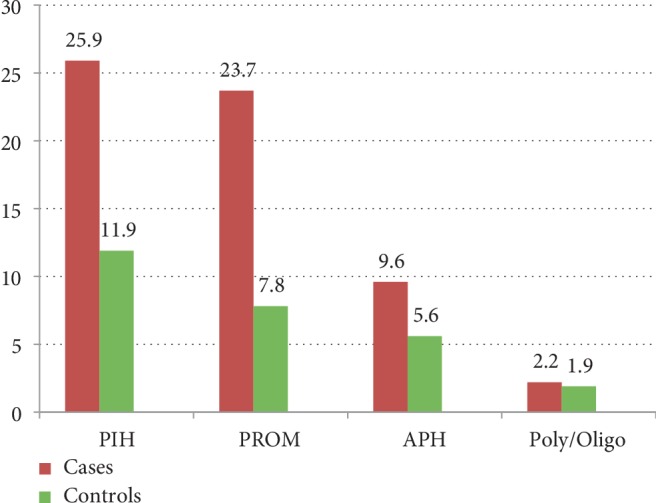
Distributions of obstetric complication in the index pregnancy among women who gave birth in Amhara region referral hospitals, Northwest Ethiopia, 2018.

More than two-fifth respondents (43.7% in cases and 55.6% in controls) were primiparous. Among Multigravida mothers, considerable participants (5.9% in cases and 3.3% in controls) had history of previous PTB and significant respondents (18.5% in case and 3.3% in controls) had history of abortion. Similarly, substantial proportion of respondents (9.6% in cases in controls) had unplanned pregnancy. Large number of participants (94.3% in cases and 93.0% in controls) had ANC follow up during the most recent pregnancy. Among mothers who had ANC visit in the indexed pregnancy, majority (60.7%) in cases and nearly one-third (29.6%) in controls had incomplete (i.e., less than four) ANC visits. And 42 (31.1%) of cases and 76 (30.3%) of controls had started their ANC visit timely (i.e. within the 1^st^16 weeks of gestation). Of multiparous mothers, about 34 (25.2%) in cases and 43 (15.9%) in controls had one or more previous history of bad obstetric complications. Majority (86.5% in cases and 91.3% in controls) of mothers had received Iron supplementation in the index pregnancy (Table 2). PIH had been reported to be the leading (25.9% in cases and 11.9% in controls) obstetric complication in the index pregnancy (Figure 1).

### 3.3. Co-Morbidities during Pregnancy and Fetal Profiles

Regarding the neonates' birth weight, the proportion of low birth weight had been observed to be 98.5% in case and 3.0% in controls. Neonatal congenital malformation had been observed in 1.5% of cases and 1.9% of controls whereas negative syphilis test result had been reported in 98.1 % of case and 99.2% of controls. MUAC ≥23 cm had been reported in nearly three-fourth (71.9%) of cases and two-third (62.2%) of controls. Most of the respondents (90.4% in cases and (95.9% in controls) had HGB level ≥11 gram/deciliter. Similarly, almost all (99.3% in cases and 99.3% in controls) mothers ≥150 cm tall. On the other way, none of the respondent had history of cigarette smoking.

### 3.4. Determinants of Preterm Birth

Findings from the bivariate logistic regression analysis show that age, residence, total family size, educational status of the women, distances from home to the nearest health facility, parity, previous history of abortion, multiple pregnancy, PROM, PIH, bad previous obstetric history and HGB level were statically associated with PTB.

However, in the multivariable logistic regression analysis; lower maternal educational status (AOR = 2.24; 95% CI: 1.28, 3.91), low HGB level (AOR = 2.75; 95% CI: 1.11, 7.31). PROM (AOR = 6.4; 95% CI: 3.23, 12.77), PIH (AOR = 4.74; 95% CI: 2.49, 9.0), abortion (AOR = 2.92; 95% CI: 1.3, 6.4) and multiple pregnancies (AOR = 4.1; 95% CI: 1.7, 9.8) had statistically significant association with PTB (Table 3).

## 4. Discussion

PTB remains a leading cause of neonatal deaths in Ethiopia where lack of reliable information on predictors of PTB is common. This study was aimed at identifying determinants of PTB among women who gave birth in referral hospitals in ANRS, northwest Ethiopia. The odds of giving PTB were higher among mothers who had HGB level <11 gm/dl, history of abortion, PROM in the index pregnancy, PIH in the index pregnancy, multiple pregnancies and no formal education.

The result of the present study showed that the odds of PTB in women with HGB level <11 gm/dl were 2.75 times higher than that in women with HGB ≥ 11 gm/dl. Similar findings have been reported in other studies conducted in Malawi [31] and East China [32]. This is also in line with studies done in different settings in Ethiopia such as Debretabor [33] and Tigray [34]. It might be explained by the biological mechanisms that how anemia, iron deficiency or both could cause preterm delivery. In fact, anemia has been identified as a risk factor for preterm delivery through several potential biological mechanisms. Accordingly, anemia (by causing hypoxia) and iron deficiency (by increasing serum nor-epinephrine concentrations) can induce maternal and fetal stress which in turn stimulates the synthesis of Corticotrophin-Releasing Hormone (CRH). In addition, Iron deficiency may also increase the risk of maternal infections which can again stimulate the production of CRH and the elevated CRH concentrations in turn are known to be a predisposing factor of PTB [35]. Thus, herein, we can deduce that prevention of anemia need to get more emphasis in the routine ANC services so as to prevent PTB.

In this study, history of abortion was found to be a significant risk factor for PTB. Accordingly, mothers who had history of abortion were 2.92 times more likely to give PTB as compared to those women who had no history of abortion. Similarly, statistically significant association between history of abortion and PTB have been observed in other studies done in Brazil [36], Iran [37] and northern Ethiopia [34]. This association is probably due to the possibility that the procedures for abortion management could cause cervical incompetency which in turn bring about PTB the subsequent pregnancies.

The present study found that the risk of PTB was 4.674 folds higher in women with PIH than that in those women with no PIH. This finding is in line with the result of studies done in Wuhan China [30], Nairobi Kenya [38], rural Kenya [39], Lagos Nigeria [40], California [41] and Brazil [42]. It is also in accordance with the findings of previous studies done in Ethiopia [12, 24, 33, 43, 44]. This finding could be explained by the existing scientific evidences which speculate that PIH is linked to vascular and placental damage which in turn induces the oxytocin receptors thereby results in PTB.

The result from multivariable regression analysis in the present study shows that multiple pregnancies have been associated with the increased likelihood of PTB. Thus, women with multiple pregnancies were 4.1 times more likely to experience PTB as compared to those women with singleton pregnancies. Similar results have been reported from other studies conduct in Brazil [36, 45], Iran [46] and Ethiopia [12, 24, 44]. The statistically significant association between multiple pregnancies and PTB might be due to the fact that multiple pregnancies could invoke uterine over distention which might revolve to end up with spontaneous PTB. In addition, multiple pregnancies are more likely to be linked with numerous complications such as preeclampsia, PROM and polyhydraminos and these all per se could contribute to iatrogenic PTB.

We found that mothers with PROM had more than six-fold increased the likelihood of having PTB as compared to mothers with no PROM. This is consistent with the study done in Nigeria [40] and Kenya [38]. It is also in accordance with the findings of local previous studies conducted in Debretabor [47], Debremarkos [48] and Jimma [24]. This could be explained by the effect of ruptured membrane on uterine contraction. Existing scientific evidences affirm that some endogenous uterotonic chemicals released when a membrane ruptured and this uterotonic chemicals in turn stimulates uterine contraction thereby causes PTB.

In the current study, the odds of giving PTB were 2.3 times higher among women who had never attended formal education than those women who had ever attended. The finding is in agreement with the report of other studies conducted in Northern Albia [49], Brazil [50] and Ethiopia [28].This could be due to the fact that improvement in education empower women so as to enhance their exposure to information, capacity in decision-making, control on the resources and confidence in dealing with family. Such empowerments in turn may help women to be aware of the recommended practices on health promotions and diseases/complication preventions. In connection to this, empirical evidences exhibit that women who had never attended formal education were less likely to have ANC follow up visits. Inadequate ANC visit again is related to PTB as studies illustrate [25, 51]. Moreover, the association can be explained by the effect of education on birth intervals that educated women are more likely to have optimal birth interval. In this regard, studies demonstrate that optimal birth interval is an independent determinant of PTB [23, 24].

## 5. Limitation of the Study

There might be a recall bias in some variables' measurement although attempts had been taken to minimize recall biases by informing the local events.

## 6. Conclusions

The odds of giving PTB was higher among women with low HGB level, no formal education, history of abortion, PIH, PROM and multiple pregnancy in the index pregnancy. Most of the determinants of PTB were found to be modifiable. Thus, putting emphasis for prevention of obstetric and gynecologic complications such as anemia, PROM and abortion would decrease the incidence of PTB. Moreover, strengthening Information Communication Education about prevention of PTB has been recommended.

## Figures and Tables

**Table 1:** Socio-demographic characteristics of study participants who gave birth in Amhara region, referral hospitals, Northwest, Ethiopia, 2018 (*n* = 405).

Variable	Cases *N* (%)	Controls *N* (%)	Total *N* (%)
*Age (Year)*			
<20	3 (2.2)	11 (4.1)	14 (3.5)
20–34	104 (77.1)	227 (84.1)	331 (81.7)
≥35	28 (20.7)	32 (11.9)	60 (14.8)

*Religion*			
Christian	130 (96.3)	249 (92.3)	379 (93.6)
Muslim	5 (3.7)	21 (7.8)	26 (6.4)

*Ethnicity*			
Amhara	131 (97)	260 (96.3)	391 (96.5)
Other^∗^	4 (3.0)	10 (3.5)	14 (3.5)

*Educational status*			
No formal education	64 (47.4)	70 (25.9)	134 (33.1)
Primary education	23 (17.0)	49 (18.1)	72 (17.8)
Secondary and above	48 (35.6)	151 (55.9)	199 (49.1)

*Spouse educational status* (*N* = 400)			
No formal education	59 (44)	71 (27)	130 (32.8)
Primary education	18 (13.4)	36 (13.5)	54 (13.4)
Secondary and above	57 (42.5)	159 (59.6)	216 (53.8)

*Spouse occupational status* (*N* = 400)			
Farmer	63 (47.0)	83 (31.2)	146 (36.5)
Merchant	15 (11.2)	58 (21.8)	73 (18.0)
Student	3 (2.3)	0	3 (0.8)
Government employee	31 (23.3)	84 (31.3)	115 (28.8)
Daily laborer	6 (4.5)	16 (6.0)	22 (5.5)
Others^∗∗^	16 (11.9)	25 (9.4)	41 (10.4)

*Occupation*			
House wife	94 (69.6)	174 (64.4)	268 (66.2)
Government employee	27 (20.0)	58 (21.5)	85 (21.0)
Merchant	7 (5.2)	17 (6.3)	24 (5.9)
Student	2 (1.5)	5 (1.9)	7 (1.7)
Daily labor	3 (2.2)	11 (4.1)	14 (3.5)
Others^∗∗∗^	2 (1.5)	5 (1.9)	7 (1.7)

^∗^Oromo and Bishangul Gumz. ^∗∗^Car driver and private employee. ^∗∗∗^Barbara shop and private employee.

**Table 2:** Obstetrics profiles of study participants who gave birth in Amhara region, referral hospitals, Northwest, Ethiopia, 2019 (*n* = 405).

Variable	Cases *N* (%)	Controls *N* (%)	Total *N* (%)
*Parity*			
1	59 (43.7)	150 (55.6)	209 (51.6)
2–5	54 (40.0)	105 (38.9)	159 (39.3)
≥5	22 (16.3)	15 (5.6)	37 (9.1)

*Types of bad obstetric history* (*n* = 94)			
Still birth	16 (20)	12 (15)	28 (35)
Early neonatal lost	11 (13.8)	9 (11.2)	20 (25.0)
APH	6 (7.2)	9 (11.2)	15 (18.7)11 (13.8)
PPH	5 (6.2)	6 (7.5)	7 (8.8)
PIH	2 (5.7)	5 (6.2)	8 (10.0)
PROM	3 (3.8)	5 (6.2)	5 (8)
OL	2 (5.9)	3 (7)	

*Onset of labor*			
Start spontaneous	103 (76.3)	214 (79.3)	317 (78.3)
Induction	23 (17.0)	29 (10.7)	52 (12.8)
CS before labor start	9 (6.7)	27 (10.0)	36 (8.9)

*Inter pregnancy interval*			
No birth interval (Primiparous)	59 (43.7)	140 (51.9)	199 (49.1)
<24 month	24 (17.8)	17 (6.3)	41 (10.1)
≥24 month	52 (38.5)	113 (41.9)	165 (40.7)

*Frequency of ANC*			
No ANC follow up	8 (5.9)	19 (7.0)80 (29.6)	27 (6.7)
<4 follow	82 (60.7)		162 (40.0)
≥4	45 (33.3)	171 (63.3)	216 (53.3)

*Duration of iron taking *(*n* = 357)			
<3 months	113 (98.3)	238 (98.3)	351 (98.3
≥3 months	2 (1.7)	4 (1.7)	6 (1.7)

*Multiple pregnancy*			
Yes	21 (15.6)	12 (4.4)	33 (8.1)
No	114 (84.4)	258 (95.6)	372 (91.9)

**Table 3:** Determinants of preterm birth among mothers who gave birth in Amhara referral hospitals, Northwest Ethiopia, 2019 (*n* = 405).

Variable	Case number (%)	Control number (%)	COR 95% CI	AOR with 95% CI
*Age (in year)*				
<20	3 (2.2)	11 (4.1)	0.5 (0.13,2.1)	0.3 (0.22, 1.27)
20–34	104 (77.1)	227 (84.1)	1	1
≥35	28 (20.7)	32 (11.9)	1.9 (1.0, 3.3)	1.53 (0.04, 1.68)

*Residences*				
Rural	71 (52.6)	95 (35.2)	2.04 (1.34, 3.11)	0.5 (0.1, 2.1)
Urban	64 (47.4)	175 (64.8)	1	1

*Educational status*				
No formal education	64 (47.4)	70 (25.9)	2.87 (1.79, 4.59)	**2.24 **(1.28, 3.91)^∗^
Primarily	23 (17.0)	49 (18.1)	1.47 (0.81, 2.67)	1.30 (0.66, 2.56)
Secondary and above	48 (35.6)	151 (55.9)	1	1

*HGB level*				
HGB < 11	13 (9.6)	11 (4.1)	2.5 (1.09,5.76)	**2.75 **(1.11, 7.31)^∗^
HGB ≥ 11	122 (90.4)	256 (95.6)	1	1

*Family size*				
≤5	114 (84.4)	254 (94.1)	2.92 (1.47, 5.81)	0.3 (0.07, 1.86)
>5	21 (15.6)	16 (5.9)	1	

*Parity*				
1	59 (43.7)	150 (55.6)	0.76 (0.49, 1.19)	0.67 (0.31, 1.26)
2–5	54 (40.0)	105 (38.9)	1	
≥5	22 (16.3)	15 (5.6)	2.85 (1.36, 5.9)	1.3 (0.2, 7.9)

*PIH*				
Yes	35 (25.9)	32 (11.9)	2.6 (1.52, 4.43)	**4.74 **(2.49, 9.0)^∗^
No	100 (74.1)	238 (88.1)	1	

*PROM*				
Yes	32 (23.7)	21 (7.8)	3.68 (2.02, 6.68)	**6.4**(3.23, 12.77)^∗^
No	103 (76.3)	249 (92.2)	1	1

*Bad obstetrics history*				
Yes	34 (25.2)	43 (15.9)	1.77 (1.07, 2.95)	1.8 (0.9, 3.7)
No	101 (74.8)	227 (84.1)	1	

*Multiple pregnancy*				
Yes	21 (15.6)	12 (4.4)	3.96 (1.88, 8.3)	**4.1 **(1.7, 9.8)^∗^
No	114 (84.4)	258 (95.6)	1	1

*Abortion*				
Yes	25 (18.5)	18 (6.7)	3.18 (1.6, 6.07)	**2.92 **(1.3, 6.4)^∗^
No	110 (81.5)	252 (93.3)	1	1

*Distances (hours)*				
<1 hour	105 (77.8)	141 (89.3)	2.3 (1.35, 4.15)	0.6 (0.4, 1.6)
≥1 hour	30 (22.2)	29 (10.7)	1	1

Bold values denote for statistical significant associations.

## Data Availability

While ethics statement has been stated, we have agreed and signed in order not to publish the raw data retrieved from information of the mothers. However, the data sets collected and analyzed for the current study is available from the corresponding author and can be obtained on a reasonable request.

## Abbreviations

ANC: Antenatal care
ANRS: Amhara national regional sate
AOR: Adjusted odds ratio
APH: Ante partum hemorrhage
CI: Confidence interval
COR: Crude odds ratio
CRH: Corticotrophin-releasing hormone
CUFYs: Children under five years
EDHS: Ethiopia demographic health survey
HGB: Hemoglobin
GA: Gestational age
HIV: Human immune deficiency virus
LMP: Last menstrual period
LNMP: Last normal menstrual period
MUAC: Mid upper arm circumference
PIH: Pregnancy induced hypertension
NM: Neonatal mortality
PROM: Premature rupture of membrane
PTB: Preterm birth
SDG: Sustainable developed goal
SPSS: Statistical package for social sciences
U/S: Ultra sound
U/S: Ultrasound
UFM: Under five mortality
WHO: World health organization.
